# Behavioral and cognitive performance of humanized *APOE*ε3/ε3 liver mice in relation to plasma apolipoprotein E levels

**DOI:** 10.1038/s41598-023-28165-3

**Published:** 2023-01-31

**Authors:** Kat Kessler, Andreas Giannisis, Greg Bial, Lander Foquet, Henrietta M. Nielsen, Jacob Raber

**Affiliations:** 1 Department of Behavioral Neuroscience, Oregon Health & Science University, Portland, OR 97239 USA; 2 Department of Biochemistry and Biophysics, Stockholm University, 10691 Stockholm, Sweden; 3 Yecuris Corporation, Tualatin, OR 97062 USA; 4 Departments of Neurology and Radiation Medicine, Division of Neuroscience, Oregon National Primate Research Center, Oregon Health & Science University, Portland, OR 97239 USA

**Keywords:** Neuroscience, Medical research, Neurology

## Abstract

Plasma apolipoprotein E levels were previously associated with the risk of developing Alzheimer’s disease (AD), levels of cerebrospinal fluid AD biomarkers, cognition and imaging brain measures. Outside the brain, the liver is the primary source of apoE and liver transplantation studies have demonstrated that liver-derived apoE does not cross the blood–brain-barrier. How hepatic apoE may be implicated in behavioral and cognitive performance is not clear. In the current study, we behaviorally tested FRGN mice with humanized liver harboring the ε3/ε3 genotype (E3-human liver (HL)) and compared their behavioral and cognitive performance with that of age-matched ε3/ε3 targeted replacement (E3-TR) mice, the latter produces human apoE3 throughout the body whereas the E3-HL mice endogenously produce human apoE3 only in the liver. We also compared the liver weights and plasma apoE levels, and assessed whether plasma apoE levels were correlated with behavioral or cognitive measures in both models. E3-HL were more active but performed cognitively worse than E3-TR mice. E3-HL mice moved more in the open field containing objects, showed higher activity levels in the Y maze, showed higher activity levels during the baseline period in the fear conditioning test than E3-TR mice, and swam faster than E3-TR mice during training to locate the visible platform in the water maze. However, E3-HL mice showed reduced spatial memory retention in the water maze and reduced fear learning and contextual and cued fear memory than E3-TR mice. Liver weights were greater in E3-HL than E3-TR mice and sex-dependent only in the latter model. Plasma apoE3 levels were similar to those found in humans and comparable in female and male E3-TR mice but higher in female E3-HL mice. Finally, we found correlations between plasma apoE levels and behavioral and cognitive measures which were predominantly model-dependent. Our study demonstrates mouse-model dependent associations between plasma apoE levels, behavior and cognition in an ‘AD-neutral’ setting and suggests that a humanized liver might be sufficient to induce mouse behavioral and cognitive phenotypes.

## Introduction

Apolipoprotein E (apoE) plays an important role in the metabolism and redistribution of lipoproteins and cholesterol. In humans, the *APOE* gene is polymorphic with three common variants (ε2, ε3 and ε4). Compared to ε3, ε4 significantly increases the risk of developing Alzheimer’s disease (AD) whereas ε2 is protective^1^. In the brain, apoE has been implicated not only in development, regeneration, neurite outgrowth, and neuroprotection, but also in pathological processes leading to neurodegeneration^2–4^. These results have led to numerous in vivo apoE studies. Importantly, rodent *Apoe*, similar to that in other mammals, is monomorphic with an amino-acid sequence resembling that of the human ε4 which is considered the ancestral variant^5^. Previous studies in human *APOE* mouse models involved expression of human apoE isoforms in brains of global murine *Apoe* deficient mice^6^. Studies with this model, as well as that of global murine *Apoe* deficient mice revealed how peripheral *APOE* deficiency, for example in the adrenal gland, might be important for behavioral phenotypes^7,8^. More recent studies involving human *APOE* targeted replacement (TR) mice expressing human *APOE* isoforms under the control of the murine *Apoe* promoter have been used to study behavioral phenotypes^9^. It is known that *Apoe* deficient mice (knock-out (KO)) exhibit an age-dependent loss of synapses and learning deficits^10^ which in part could be alleviated by restoration of plasma apoE^11^.

Outside the brain, the liver is the primary source of apoE and accounts for more than 90% of the plasma apoE levels^12^. Liver transplantation studies involving engraftment of a liver with a non-endogenous *APOE* genotype resulted in the plasma apoE phenotype of the donor but a brain apoE phenotype of the recipient^13^, indicating that liver-derived plasma apoE does not cross the BBB. Nevertheless, plasma apoE levels in clinical studies were shown to be associated with the risk of developing AD and other types of dementia^14^. We and others have shown that plasma but not cerebrospinal fluid (CSF) apoE levels are *APOE* genotype-dependent^15–17^, with ε4-carriers exhibiting the lowest levels. Furthermore, plasma apoE levels were also found associated with CSF AD biomarkers, cognition, cerebral gray matter volume and glucose metabolism^17,18^. Yet, since plasma apoE does not cross the BBB, plasma apoE has long been dismissed as irrelevant to neuropathological processes. To assess whether specifically liver-derived apoE might affect the brain, we recently compared the brains of *Fah−/−*, *Rag2−/−, Il2rg−/− *mice on the non-obese diabetic (NOD) background (FRGN) with humanized-livers (through transplantation with primary human hepatocytes) of an *APOE* ε4/ε4 versus an *APOE* ε2/ε3 genotype. Compared to *APOE* ε2/ε3 liver mice, endogenous mouse apoE levels were lower in the brains of *APOE* ε4/ε4 liver mice and the liver *APOE* ε4/ε4 genotype associated with detrimental changes in markers of synaptic integrity, neuroinflammation and insulin signaling^19^. Furthermore, plasma apoE4 levels were consistently associated with negative outcomes of the marker analyses^19^. Consistent with a role of the liver in brain phenotypes is the neurodegenerative phenotype described in mice that generate human amyloid β (Aβ) solely in the liver^20^, altered levels of Aβ-degrading enzymes in the livers of AD patients^21^, and the association between altered liver enzymes with an AD diagnosis, cognition, neuroimaging measures, and cerebrospinal fluid biomarkers^22^. A role of the liver in brain phenotypes is also consistent with alterations in the gut microbiome and the development of neurodegenerative conditions, including Parkinson disease^23,24^ and AD^25,26^ that likely involve the bi-directional gut-liver-brain axis^27,28^.

In the current study, we behaviorally tested male and female FRGN mice with humanized ‘AD-neutral’ ε3/ε3 livers (E3-HL) and compared their behavioral and cognitive performance to that of age-matched ε3/ε3 TR mice (E3-TR) with the overall aim to assess whether plasma apoE levels were correlated with behavioral or cognitive measures, and liver size in both models. We focused our analysis on hippocampus-dependent spatial habituation in the open field^29^, object recognition with a 24 h interval between training and testing^30^, spontaneous alternation^31^ in the Y maze, novel arm measures in the spatial Y maze^32^, spatial learning and memory in the water maze^33^, and contextual fear conditioning^34^. As alterations in activity or anxiety measures can affect performance on cognitive tests, we assessed both parameters in the open field test. To determine whether changes might be specific to hippocampus-dependent memory, we assessed hippocampus-independent cued fear memory^35^ as well. To assess the possible relationships between the distinct behavioral and cognitive measures, we performed a principal components analysis. We included plasma apoE levels and liver size as outcome measures to determine whether they load on the same factors as the behavioral and cognitive measures, supporting that they contribute to them.

## Methods

All methods were carried out in accordance with relevant guidelines and regulations.

### Animals

In this study, we behaviorally tested 19 E3-HL (FRG YF-10) (9 female,10 male) mice which through transplantation with primary human hepatocytes at the age of 2 months acquire highly humanized livers (70–95%)^19,36^ (Yecuris, Hillsboro, Oregon). The sex of the donor and the recipient mice were matched. Two donors were used for the current study, one female donor and one male donor. Both donors were younger than 19 years of age. The mice were maintained on a custom-formulated FRG YF-10 diet, ad libitum, made in an ISO 9001 facility based on a modified Baker synthetic rodent diet^37^. The drinking water was supplemented with dextrose or sulfamethoxazole/trimethoprim (SMX/TMP), using 8-day intervals between the two drinking solutions. The mice were shipped from Yecuris to OHSU following quarantine. We also tested 20 age-matched E3-TR mice (10 mice per sex), a mouse model generated by Dr. Sullivan^38^, maintained on PicoLab Rodent Diet 20, no. 5053; PMI Nutrition International, St. Louis, MO, United States). Food and water were provided ad libitum and lights were on a standard 12 h light: dark cycle. Like for the E3-HL mice, the drinking water of the E3-TR mice was also supplemented with dextrose or sulfamethoxazole/trimethoprim (SMX/TMP) prior to and during behavioral testing, using 8-day intervals between the two drinking solutions. All mice were housed and tested in the same room under the same environmental conditions besides the difference in diet indicated above. The behavioral testing of the mice was performed at 7 months of age. All animal procedures were reviewed and approved by the OHSU IACUC and in accordance with AAALAC standards. All procedures followed the ARRIVE guidelines. Researchers were blinded to the sex of the mice throughout the duration of experiments.

### Behavioral and cognitive testing

Behavioral and cognitive testing was conducted and the data analyzed by an experimenter blinded to the sex of the mice. The group code was broken once the data were analyzed. Procedures complied with the NIH Guide for the Care and Use of Laboratory Animals and with IACUC approval at OHSU. Behavioral and cognitive testing of the mice was conducted in the following order: open field (days 1 and 2) and novel object recognition (days 3 and 4) in week 1; spatial learning and memory in the water maze (days 1–6) in week 2; and spontaneous alternation in the Y maze (day 1), the spatial version of the Y maze with 24 h between training and testing (days 2 and 3), and contextual and cued fear conditioning (days 4 and 5) in Week 3. Between trials of each test in the recipient mice, all testing surfaces were cleaned with a 70% ethanol solution to eliminate odor cues unless indicated otherwise. The open field and novel object recognition, water maze, Y maze, spatial Y maze and fear conditioning tests were performed as described^25,26^. The mice were euthanized on day 2 of Week 4 of the testing. Plasma was collected and stored for analysis as described in detail below.

### Open field and novel object recognition

Exploratory activity and anxiety were measured in the open field. In this task, mice were placed in enclosures (L 40.6 × W 40.6 × H 40.6 cm) for a 10-min trial on 2 consecutive days, as described^39^. Time spent in the center of the open field was analyzed to assess measures of anxiety. The following day, two identical objects were placed in the open field and mice were allowed to explore for a 15-min trial. The objects were placed 10 cm apart and 15 cm from the adjacent walls of the arena. The next day, one object was replaced with a novel object and mice were allowed to explore the open field for 15 min. During object recognition trials, the objects were affixed to the floor of the arena using masking tape. Physical interaction with the object in the form of sniffing within a 2 cm proximity was coded as object exploration. Performance of mice was tracked using Ethovision 14 XT video tracking software. Videos were later hand scored to analyze object exploration. Mice exploring the objects for less than 10 s were removed from the analysis. The discrimination index, defined as the time spent exploring the novel object minus the time spent exploring the familiar object divided by the time spent exploring both objects was calculated for each mouse to assess object recognition.

### Water maze

Hippocampus-dependent spatial learning and memory was assessed in the water maze^33^. The maze consisted of a circular pool (diameter 140 cm), filled with opaque water (24 °C), divided conceptually into four quadrants. Mice were first trained to locate an “escape” platform (plexiglass circle, 6 cm radius) submerged 2 cm below the surface of the water and made visible by the use of a cue (a colored cylinder, 2.5 cm radius, 8 cm height) during the “visible platform” trials (days 1 and 2). For the visible platform training days, there were two daily sessions, morning and afternoon, which were separated by an intersession interval of 2 h. Each session consisted of two trials, with 10-min inter-trial intervals. Mice were placed into the water facing the edge of the pool in one of nine randomized locations (consistent for each mouse). A trial ended when the mouse located the platform. Mice that failed to locate the platform within 60 s were led to the platform by placing a finger in front of their swim path. Mice were taken out of the pool after they remained on the platform for a minimum of 10 s.

During the visible platform sessions, the location of the platform was moved between each of the four quadrants to avoid procedural biases in task learning. Subsequent to the visual trials, mice were trained to locate a hidden platform, requiring the mice to rely on extra maze cues for spatial reference and orientation. The platform was not rotated during the hidden platform trials and remained in the same location. Twenty-four hours after the last hidden platform training trial, spatial memory retention of the mice was assessed in a “probe” trial (no platform). During the probe trial, mice were placed into the water in the quadrant opposite of the target quadrant. The time spent in the target quadrant compared to the time spent in the three non-target quadrants was analyzed. In addition, cumulative distance to the target location, latency to first platform crossing, and number of platform crossings were analyzed as performance measures.

The swimming patterns of the mice were recorded with Noldus Ethovision video tracking software (Ethovision XT, Noldus Information Technology, Wageningen, Netherlands) set at six samples/s. The time to locate the platform (latency) and cumulative distance to the platform location were used as measures of performance for the visible and hidden platform sessions. Latency to reach the target was measured in seconds, and was calculated for each day by averaging values from the six daily trials. Because swim speeds can influence the time it takes to reach the platform, they were also analyzed.

### Y maze

Activity levels and hippocampus-dependent spontaneous alternations were assessed in the Y-maze as previously described^40^. The Y-shaped maze (O’ Hara & Co., Ltd, Tokyo, Japan) had raised sides (3.8 cm bottom width, 12.55 cm top width, 12.55 cm height) with plastic, opaque grey arms (37.98 cm length). Mice were placed in the center of the maze at the beginning of a 5-min trial. Performance of the mice was tracked using Ethovision 14 XT video tracking software (Wageningen, the Netherlands). Digital videos were later analyzed to measure the number of arm entries and to calculate the percent spontaneous alternations. The criteria for an arm entry was when all four limbs were within the arm. The spontaneous alternation percentage was calculated by dividing the number of 3-arm alternations by the number of possible 3-arm alternations and multiplying the value by 100.

### Spatial Y maze

The spatial Y-maze test and fear conditioning tests were performed as described^25,26^. One arm was blocked off and mice were allowed to explore the maze for 15 min on day 1. All of the arms were accessible on day 2, and mice were allowed to explore for 5-min. Performance measures were the number of entries into and the percent time spent in the novel arm (the arm that was blocked off on day 1). An arm entry was defined when all four limbs were within the arm.

### Fear conditioning

Mice were trained and tested for fear learning and memory using a Med Associates mouse fear conditioning system and VideoFreeze automated scoring system (Med Associates, St. Albans, Vermont), as described in detail^25,26^ and validated^41^. On day 1, the mice were placed inside a dark fear-conditioning chamber. The chamber lights (100 lx) were turned on at zero seconds, followed by a 90-s habituation period and a subsequent 30-s (2800 Hz, 80 dB) tone (cue). A 2-s 0.5 mA foot shock was administered at 28 s, co-terminating with the tone at 30 s. After a 60 s inter-stimulus-interval, there was another tone-shock pairing. On day 2, contextual fear memory was assessed during re-exposure to the training environment for 300 s. Three hours later, mice were exposed to a new environment (scented with vanilla extract, cleaned with 10% isopropanol instead of 0.5% glacial acetic acid, novel floor texture covering the shock-grid, and rounded walls). The mice were habituated to the new environment for 90 s (pre-tone), and exposed to the tone (cue) for 180 s. Performance measures were motion during the test phases (based on the VideoFreeze automated scoring system software and expressed in arbitrary units as the size of the enclosure is not calibrated with this software since the camera is positioned on a wall and not from above), percent time spent freezing in response to the contextual environment or the tone, and motion during the shock (to account for potential differences in response to the shock during fear learning).

### Liver weights and plasma apoE analyses

Following behavioral and cognitive testing, the mice were killed by cervical dislocation and blood was collected in EDTA-containing tubes. The tubes were centrifugated to obtain plasma and stored at −80 °C. The liver was dissected as well and shipped on dry ice along with the plasma samples to the Nielsen laboratory at Stockholm University for analyses. The liver weights were determined and apoE levels in the plasma of the mice analyzed as described^19,42,43^. Briefly, one 96 well plate was coated with the mouse monoclonal pan-apoE antibody WUE-4 (Novus Biologicals) diluted in 0.05 M sodium carbonate buffer (NaN_3_, NaHCO_3_, Na_2_CO3, pH 9.6) to a final concentration 1 μg/mL and incubated overnight at room temperature. The next day, the wells were washed with phosphate buffered saline (PBS, pH 7.4, VWR) containing 0.05% Tween^®^20 (VWR), blocked with 1% w/v nonfat dried milk powder (NFDMP, PanReac AppliChem) in PBS, then washed again and finally incubated with plasma samples from E3-HL (*n* = 17) or E3-TR mice (*n* = 20), previously diluted 1:4000 or 1:2000 respectively in blocking solution. Plasma samples were applied in a random order and blinded to the experimenter. Following sample incubation, apoE was captured using a biotinylated pan-apoE antibody (Meridian Lifesciences) diluted to 0.2 μg/mL in blocking solution and detected using the enzymatic reaction between 0.1 μg/mL horse radish peroxidase (HRP) conjugated streptavidin (Fitzgerald) in 1% NFDMP/PBS and the substrate tetramethylbenzidine (TMB, Sigma-Aldrich). Reaction was stopped by adding 1 M sulfuric acid and the absorbance was measured at 450 nm with the HiPo-96 microplate photometer (BIOSAN, Riga, Latvia). Levels of plasma apoE3 were determined by the use of a calibration curve generated by serial dilutions of apoE3 recombinant protein (Fitzgerald) in blocking buffer. Samples and calibrator points were run in duplicates and the obtained densities were averaged.

### Assessment of plasma apoE distribution in plasma lipoparticles

In order to assess the distribution of plasma apoE in the circulating very low density lipoproteins (VLDL), low density lipoproteins (LDL) and high density lipoproteins (HDL), we employed a strategy to fractionate plasma from both mouse models by use of size exclusion chromatography (SEC) whereafter apoE levels in the lipoprotein fractions were determined by ELISA. Due to limited sample volume the experiment was performed by use of plasma pools generated by mixing equal amounts of plasma from either all female or all male E3-HL versus E3-TR mice (four pools in total). Plasma pools were fractionated on a Superose 6 Increase 10/300 GL (GE Healthcare Life Sciences) column attached to an ÄΚΤΑ pure 25 fast protein liquid chromatography (FPLC) instrument (GE Healthcare Life Sciences). Prior to fractionation each pool was diluted two-fold with elution buffer (50 mM sodium phosphate, 150 mM sodium chloride, 1 mM EDTA and 0.02% sodium azide, pH 7.4), centrifuged at 10,000×*g*, for 5 min at 4 °C whereafter 150 μL of diluted sample were loaded on the SEC column. Proteins were eluted with 1.5 column volumes (CV) of the elution buffer at a flow rate of 0.4 mL/min as previously described^44^, and 60 fractions of 0.5 mL each, were collected (Fig. 8A–C). The resulting fractions were pooled into six pools with a final volume of 1 mL, based on the size of the eluted proteins and their correspondence to the elution profile of purified plasma VLDL, LDL and HDL (Sigma-Adrich/Merck). The first fraction pool was generated from the first four eluted fractions with a molecular weight higher than VLDL. The second, third and fourth fractions were obtained by pooling of fractions 5–12 (Pool 2), 13–19 (Pool 3) and 20–22 (Pool 4) corresponding to the size of VLDL, LDL and HDL eluted fractions. The fifth fraction pool contained proteins of size ranging between 5 and 99 kDa (fractions 23–30), and the sixth pool included proteins with a size less than 5 kDa (fractions 31–60). The amount of apoE in all the fraction pools was determined by use of ELISA as described in the previous section. The distribution of lipoprotein-associated apoE [percentage (%)] in the different lipoprotein fractions [VLDL (fraction pool 2), LDL (fraction pool 3) and HDL (fraction pool 4)] was determined by dividing the amount of apoE (μg) in each lipoprotein fraction with the total amount of lipoprotein-bound apoE (the total apoE combined in the three lipoprotein fractions) multiplied by 100.

The size of the eluted proteins was determined using gel filtration standards ranging from 1.35 to 670 kDa (Bio-Rad) whereas the elution profile of VLDL, LDL and HDL was assessed using human plasma isolated lipoproteins (Sigma-Aldrich/Merck). Both the standards and purified lipoproteins were applied under the same conditions as the samples. The experimental procedure was carried out at 4 °C. Raw chromatographic spectra were generated and analyzed by Unicorn 7.5 (GE Healthcare Life Sciences).

### Statistical analysis

Data are expressed as mean ± SEM. Behavioral and cognitive performance measures were analyzed using ANOVAs or repeated-measures ANOVAs with model (i.e., HL or TR) and sex as factors. In the case of repeated-measures ANOVA, sphericity was tested and Greenhouse–Geisser corrections were used when appropriate. For the percent time spent exploring the novel and familiar objects, paired *t*-tests were used. Statistical significance was determined using an error probability level of *p* < 0.05. Correlations between plasma apoE levels and behavioral and cognitive measures in E3-HL and E3-TR mice were analyzed using the Pearson Correlation test and the Spearman Rank correlation test, respective, as appropriate based on inspection of the distribution of the data, as well as partial correlations (*r*(degree of freedoms) controlling for sex. Data were analyzed using SPSS Statistics for Windows (Version 25, Armonk, NY: IBM Corp., Chicago, IL; https://www.ibm.com/products/spss-statistics), JMP statistical software version 16.1.0 (SAS Institute, NC, USA; https://www.jmp.com/en_se/home.html) and GraphPad Prism software, version 9.2 (San Diego, CA; https://www.graphpad.com/).

To determine whether measures of anxiety were correlated with cognitive measures, we assessed correlations between percent time spent in the center of the open field with cognitive measures. To determine whether activity levels were correlated with cognitive measures, we assessed correlations between distance moved on the first day of open field testing with cognitive measures. Correlation analyse were performed using the Pearson Correlation test.

A principal components factor analysis (PCA) was performed to determine the relationship between performances on the various behavioral tasks and between performance on the various behavioral tasks, plasma apoE and liver weight on the level of individual mice. The analysis was undertaken two reasons: (1) to determine whether plasma apoE or liver weight might contribute significantly to performance on behavioral tasks and load on the same factor(s), and (2) to approximate to what extent the distinct measures assess the same underlying abilities in the mice. Measures entered into the model were: distance moved on the first and second day in the open field (activity measure); time spent in the center of the open field (anxiety measure); distance moved in the open field containing objects; discrimination index on the object recognition test (object recognition); mean time to locate the visible platform in the water maze; mean time to locate the hidden platform in the water maze; mean swim speed during the visible platform training in the water maze; percent time in the target quadrant, cumulative distance to the platform location, latency to locate the platform location, and crosses over the platform location in the water maze probe trial; baseline motion prior to the first tone during fear learning in the fear conditioning test; percent freezing in the contextual fear memory test; percent freezing in the cued fear memory test; distance moved and spontaneous alternation in the Y maze test; percent time in the novel arm and percent entries in the novel arm in the 24 h Y maze test; plasma apoE levels; and liver weight. The PCA analysis was performed using SPSS, and factors with eigenvalue > 1.0 were considered significant. The verimax rotated matrix was used to interpret the factor loadings.

### Ethics approval

The animal study was reviewed and approved by the OHSU IACUC Committee.

## Results

### Open field and novel object recognition

**Figure 1:**
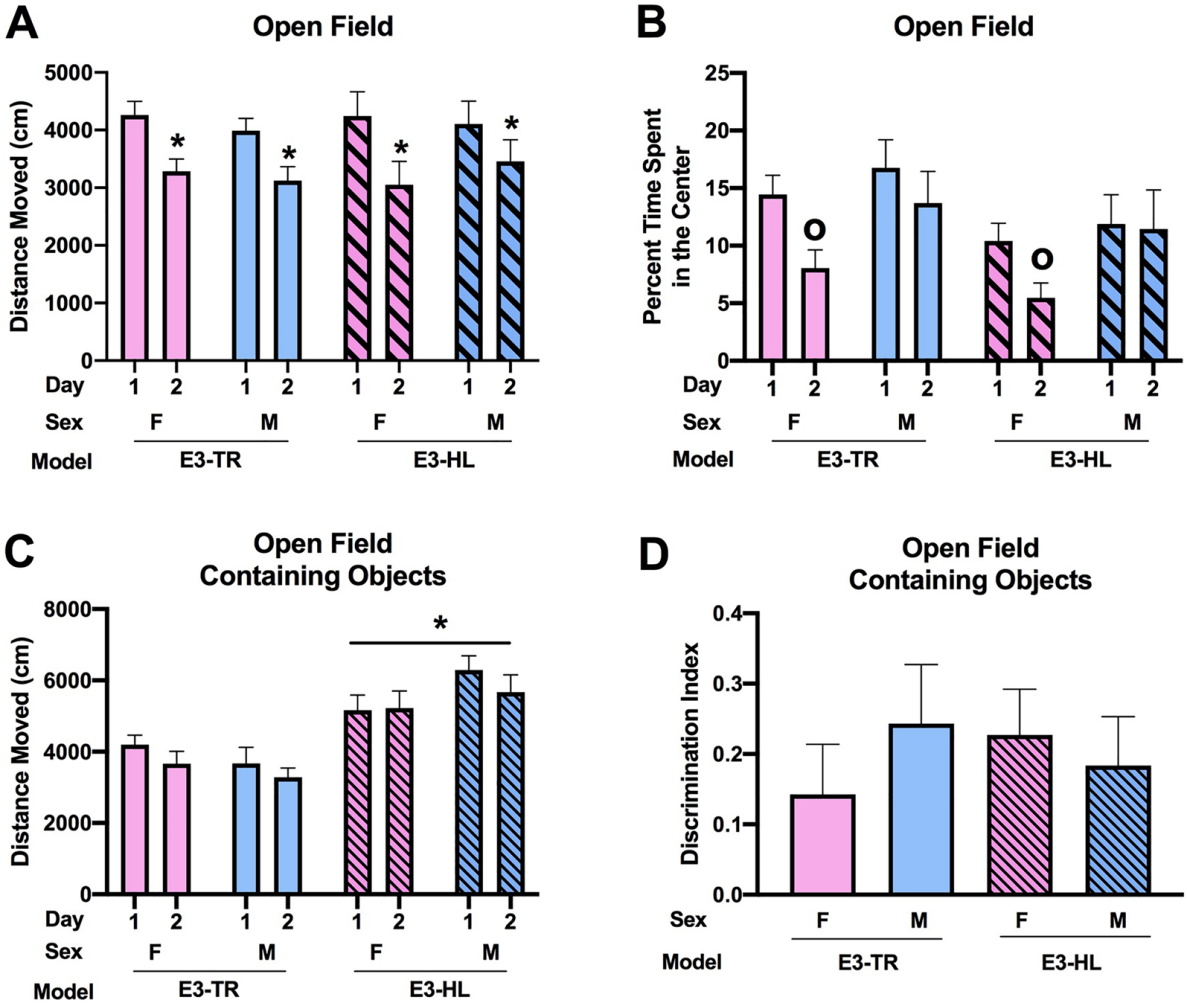
**(A)** All groups showed spatial habituation learning and moved less on day 2 than day 1. *p < 0.001). (**B)** There was a day x sex interaction for time spent in the center of the open field (*F*^1,35^ = 4.998, *p* = 0.032). In females, there was an effect of day with females spending less time spent in the center of the open field on day 2 than day 1. ^o^*p* = 0.003. (**C)** In the open field containing objects, E3-HL mice moved more than E3-TR mice. *p < 0.001. (**D)** Object recognition was comparable in all groups. The behavioral testing of the mice was performed at 7 months of age.

In the open field, all groups (female and male E3-HL and E3-TR mice) showed spatial habituation learning and moved less on day 2 than day 1 (effect of day: (*F*(1, 35) = 85.059, *p* < 0.001) (Fig. 1A). There was no effect of model or sex. When the time spent in the more anxiety-provoking center of the open field was analyzed, there was an effect of day (*F*(1, 35) = 17.936, *p* < 0.001) and a day × sex (*F*(1, 35) = 4.998, *p* = 0.032) interaction (Fig. 1B). In females, there was an effect of day with females spending less time in the center of the open field on day 2 than day 1 (*F*(1, 17) = 20.28, *p* = 0.003) and a trend towards an effect of model with a trend towards E3-HL mice spending less time in the center of the open field than E3-TR mice (*F*(1, 17) = 3.491, *p* = 0.079). This was sex-dependent and not seen in males.

Next, activity levels in the open field containing objects were analyzed. E3-HL mice moved more in the presence of objects than E3-TR mice (*F*(1, 35) = 27.035, *p* < 0.001) (Fig. 1C). There was no effect of model or sex on the discrimination index, indicating comparable object recognition in all groups (Fig. 1D).

### Water maze

**Figure 2:**
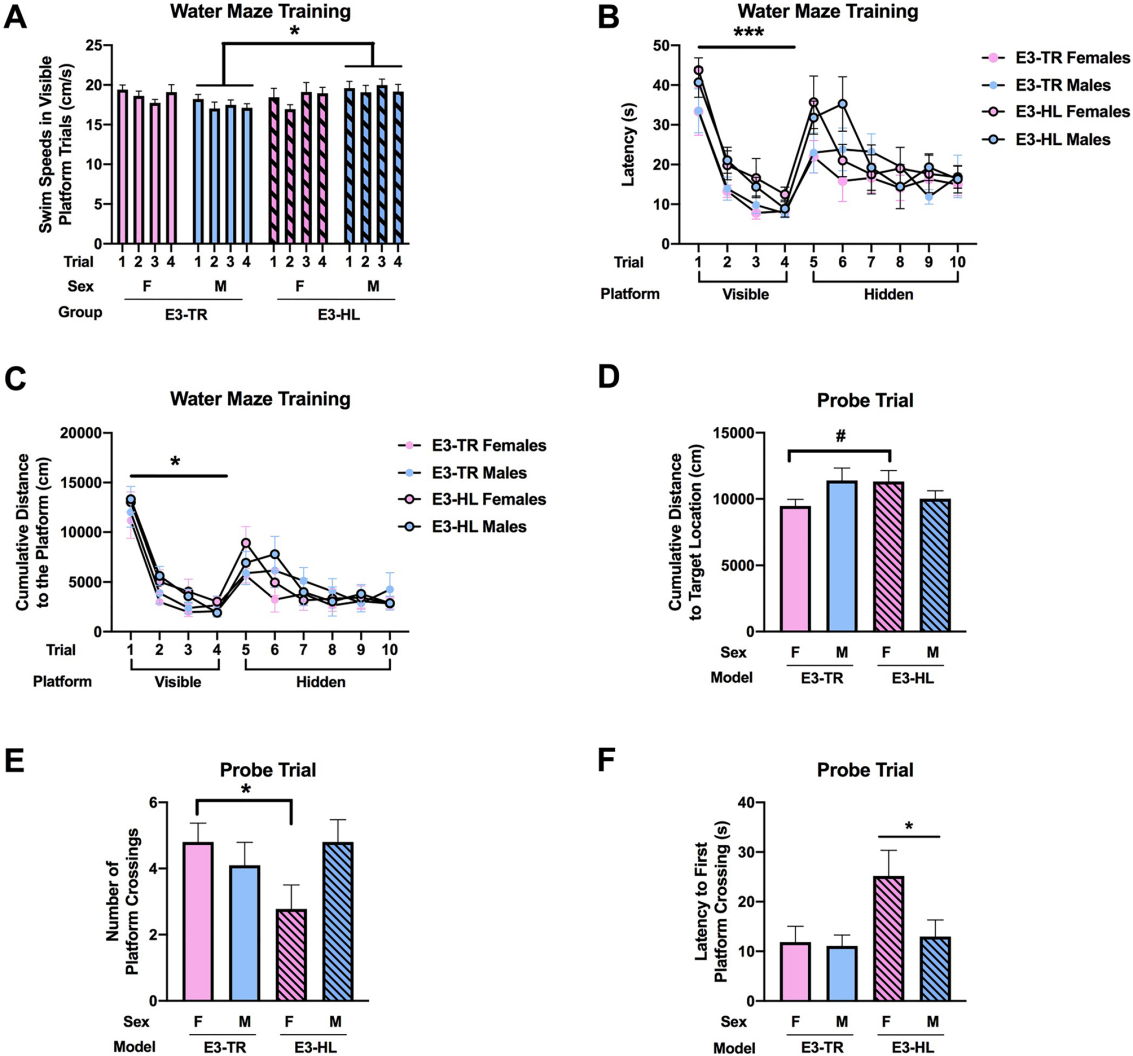
**(A)** During training to locate a visible platform in the water maze, E3-HL male mice swam faster than E3-TR male mice. *p = 0.015. (**B)** During visible platform training, E3-TR mice located the visible platform faster than E3-HL mice. ***p = 0.001. (**C)** During visible platform training, E3-TR mice showed a lower cumulative distance to the platform location that E3-HL mice. *p = 0.023. (**D)** In the probe trial (platform removed), there was a model × sex interaction for cumulative distance to the target location. There was a trend towards a greater cumulative distance to the target location in E3-HL than E3-TR females. ^#^*p* = 0.0651. (**E)** There was also a model x sex interaction for the number of crosses over the platform location. In females, E3-HL mice crossed the platform location less than E3-TR mice. *p = 0.0408. (**F)** The latency to first reach the platform location was higher in E3-HL than E3-TR mice. *p = 0.039. The behavioral testing of the mice was performed at 7 months of age.

During training to locate a visible platform in the water maze, there was a model × sex interaction (*F*(1, 35) = 4.363, *p* = 0.044) (Fig. 2A). In males, there was an effect of model (*F*(1, 18) = 7.200, *p* = 0.015), with E3-HL mice swimming faster than E3-TR mice. In contrast, there was no effect of model on swim speeds during the visible platform training in females.

E3-TR showed better task learning than E3-HL mice. E3-TR mice located the visible platform faster (*F*(1, 35) = 12.066, *p* = 0.001) (Fig. 2B) and showed a lower cumulative distance to the platform location (*F*(1, 35) = 5.632, *p* = 0.023) (Fig. 2C) than E3-HL mice. All groups learned to locate the visible platform and improved their performance with training (latency: effect of trial: (*F*(2.307, 80.749) = 66.885, *p* < 0.001 (Fig. 2B); cumulative distance to the platform location: effect of trial: (*F*(2.217, 77.606) = 94.595, *p* < 0.001 (Fig. 2C)).

During training to locate the hidden platform in the water maze, all groups improved their performance with training (latency: effect of trial: (*F*(5, 175) = 5.497, *p* < 0.001 (Fig. 2B); cumulative distance to the platform location: effect of trial: [*F*(5, 175) = 8.358, *p* < 0.001 (Fig. 2C)] but there were no effects of model or sex.

When spatial memory retention was assessed in the probe trial (platform removed), there was a model × sex interaction for cumulative distance to the target location (*F*(1, 35) = 4.805, *p* = 0.035) (Fig. 2D). There was a trend towards a greater cumulative distance to the target location in E3-HL than E3-TR females (*t* = 1.972, *p* = 0.0651). There was also a model × sex interaction for the number of crosses over the platform location (*F*(1, 35) = 4.160, *p* = 0.049) (Fig. 2E). In females, E3-HL mice crossed the platform location less than E3-TR mice (*t* = 2.214, *p* = 0.0408). Finally, there was an effect of model for latency to cross the platform location for the first time (*F*(1, 35) = 4.602, *p* = 0.039) (Fig. 2F). The latency to first reach the platform location was higher in E3-HL than E3-TR mice. This seems driven by the HL-E3 female mice. These patterns were sex-dependent and not seen in males.

### Y maze

**Figure 3:**
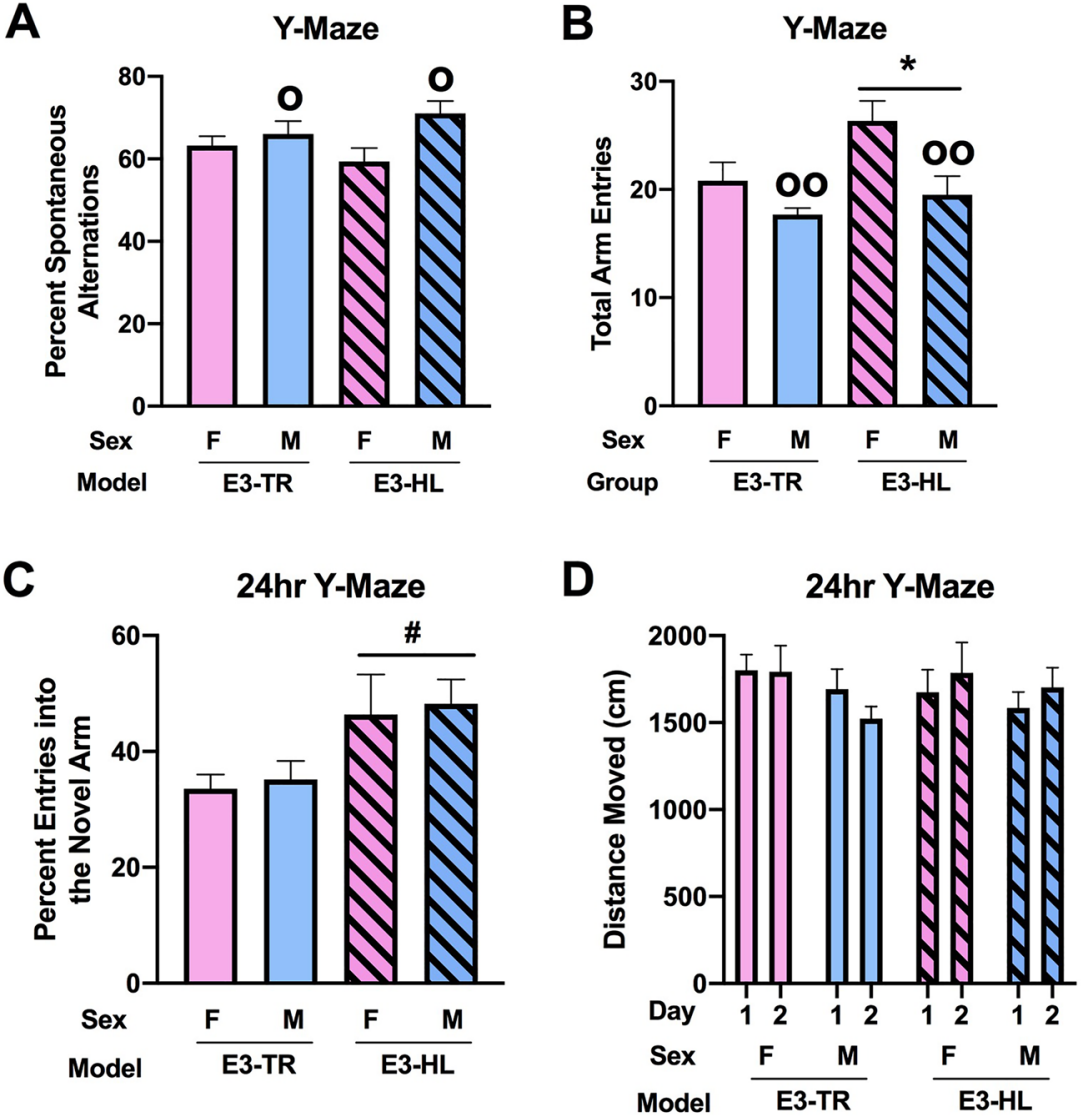
**(A)** Males showed a higher percent spontaneous alternation than females. ^o^*p* = 0.018. (**B)** E3-HL showed more arm entries than E3-TR mice. *p = 0.023. Females showed more arm entries than males. ^oo^*p* = 0.003. (**C)** There was a trend towards a higher percent entries of E3-HL than E3-TR mice in the novel arm of the spatial Y maze. ^#^*p* = 0.070. (**D)** There was a trend towards a model × day interaction for activity levels in the spatial Y maze (*F*(1, 35) = 3.547, *p* = 0.068). The behavioral testing of the mice was performed at 7 months of age.

When hippocampus-dependent spontaneous alternation was assessed, males showed a higher percent spontaneous alternation than females (*F*(1, 35) = 6.127, *p* = 0.018) (Fig. 3A). There was an effect of model on arm entries with more arm entries in E3-HL than E3-TR mice (*F*(1, 35) = 5.665, *p* = 0.023) (Fig. 3B). There was also an effect of sex on arm entries in the Y maze with more arm entries in females than males (*F*(1, 35) = 10.394, *p* = 0.003) (Fig. 3B). When instead of arm entries the total distance moved was used as the activity measure, the pattern was the same but with an effect of sex (*F*(1, 35) = 10.62, *p* = 0.0041) but not of the model (Suppl. Fig. 2).

### Spatial Y maze

Next, a different enclosure was used to assess hippocampal function in the spatial Y maze, involving a 24-h interval between training/learning and memory retention. There was a trend towards an effect of model on the percent entries in the novel arm with a trend towards a higher percent entries of E3-HL than E3-TR mice in the novel arm (*F*(1, 35) = 3.506, *p* = 0.070) (Fig. 3C). When activity levels were assessed in the spatial Y maze, there was a trend towards a model × day interaction (*F*(1, 35) = 3.547, *p* = 0.068) (Fig. 3D).

### Fear conditioning

**Figure 4:**
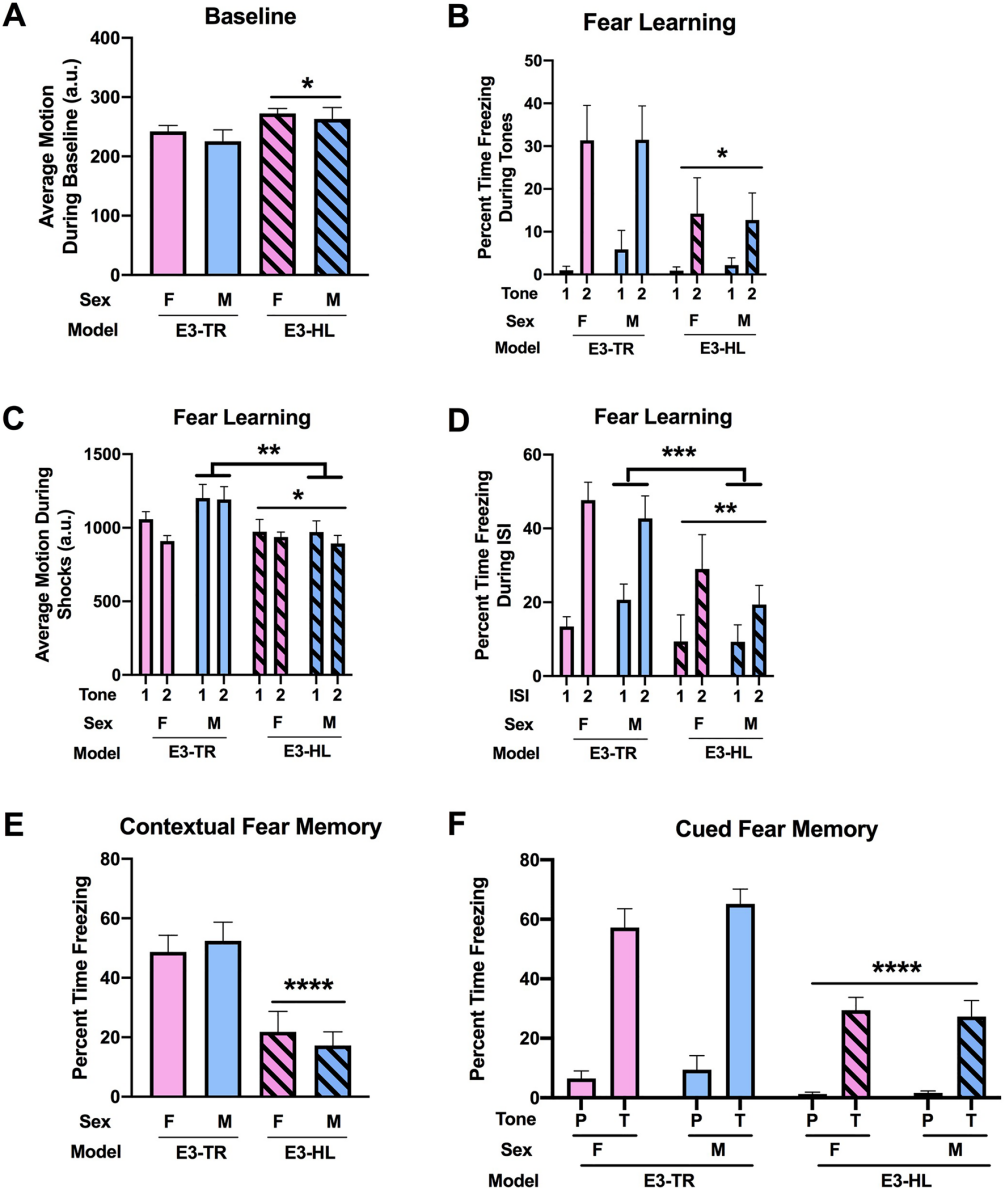
**(A)** During fear learning, prior to the first tone, E3-HL mice showed higher activity levels than E3-TR mice. *p = 0.035). (**B)** Freezing levels during the tones were lower in E3-HL than E3-TR mice. *p = 0.037. (**C)** The response to the shocks was lower in E3-HL than E3-TR mice. *p = 0.011. In males, there was also a lower response to the shocks in E3-HL than E3-TR mice **p = 0.0087. (**D)** Freezing levels between the tone-shock intervals were lower in E3-HL than E3-TR mice. **p = 0.006. In males, freezing levels during the ISIs were lower in HL-E3 than E3-TR mice. *** *p* = 0.0017. (**E)** During the contextual fear memory test, freezing levels were lower in E3-HL than E3-TR mice. ****p < 0.001. (**F)** In the cued fear memory, freezing levels were also lower in E3-HL than E3-TR mice. ****p < 0.001. The behavioral testing of the mice was performed at 7 months of age.

During fear learning, prior to the first tone, E3-HL mice showed higher activity levels than E3-TR mice (*F*(1, 35) = 4.790, *p* = 0.035) (Fig. 4A). When freezing during the tones was analyzed, there was an effect of model, with lower freezing levels in E3-HL than E3-TR mice (*F*(1, 35) = 4.719, *p* = 0.037), an effect of tone with higher freezing levels during the second than first tone (*F*(1, 35) = 32.207, *p* < 0.001) and a model × tone interaction (*F*(1, 35) = 5.209, *p* = 0.029) (Fig. 4B). When the response to the shocks was analyzed, there was a main effect of model with a lower response in E3-HL than E3-TR mice (*F*(1, 35) = 7.271, *p* = 0.011) and a model × sex interaction (*F*(1, 35) = 4.673, *p* = 0.038) (Fig. 4C). In males, there was an effect of model with a lower response in E3-HL than E3-TR mice (*F*(1, 18) = 8.673, *p* = 0.0087). Next percent time freezing between the tone-shock intervals, a measure of fear learning, was analyzed. There was an effect of model with lower freezing levels in E3-HL than E3-TR mice (*F*(1, 35) = 8.430, *p* = 0.006), an effect of ISI with higher freezing levels during the second than first ISI (*F*(1, 35) = 59.027, *p* < 0.001), and a model × ISI interaction (*F*(1, 35) = 5.604, *p* = 0.024) (Fig. 4D). In males, there was an effect of model with lower freezing levels during the ISIs in HL-E3 than E3-TR mice (*F*(1, 36) = 11.57, *p* = 0.0017). This pattern was not seen in female mice.

When hippocampus-dependent contextual fear memory was assessed the following day, there was an effect of model with lower freezing levels in E3-HL than E3-TR mice (*F*(1, 35) = 28.145, *p* < 0.001) (Fig. 4E). When hippocampus-independent cued fear memory was assessed, there also was an effect of model with lower freezing levels in E3-HL than E3-TR mice (*F*(1, 35) = 24.144, p < 0.001) (Fig. 4F).

### Liver weights and plasma apoE levels

**Figure 5:**
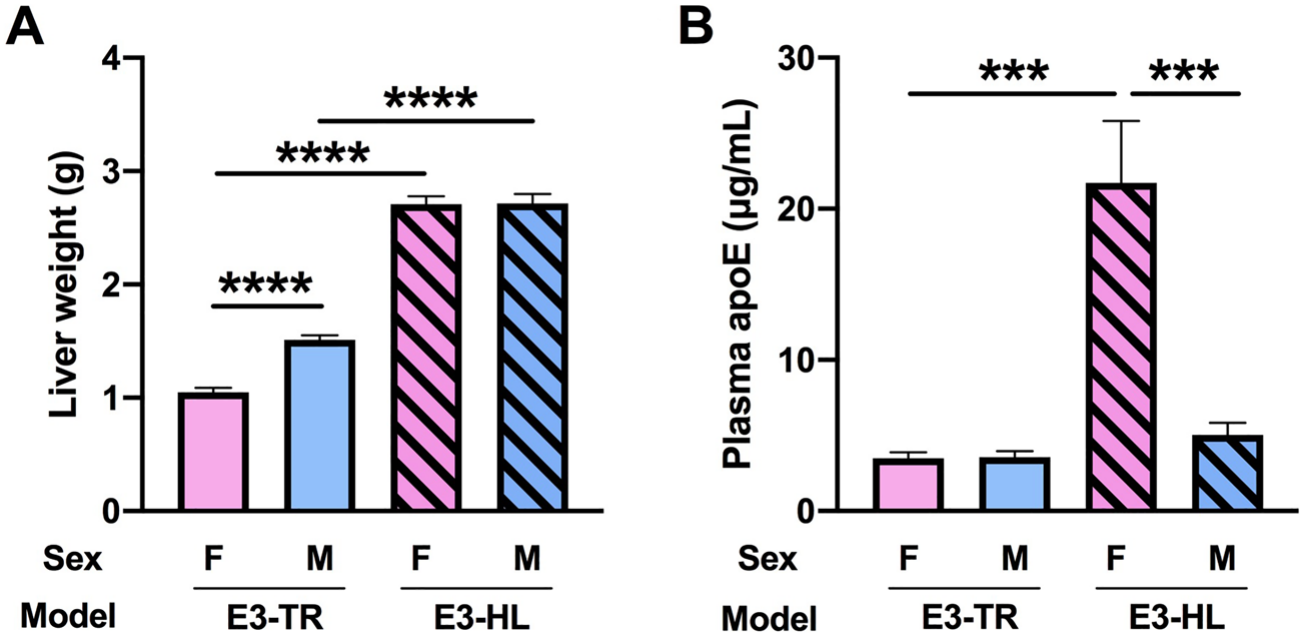
**(A)** Liver weights were greater in E3-HL than E3-TR mice. In addition, liver weights were greater in male versus female E3-TR mice. ****p < 0.0001. (**B)** Plasma apoE levels were fourfold higher in E3-HL female versus E3-HL male or E3-TR female mice. ***p < 0.005.

We found effects of model (*F*(1, 35) = 536.8995, *p* < 0.0001) and sex (*F*(1, 35) = 14.6201, *p* < 0.0005) on liver weights and there was also a sex × model interaction (*F*(1, 35) = 13.7523, *p* < 0.0007) (Fig. 5A). Liver weights were greater in E3-HL than in E3-TR mice. In addition, while liver weights were comparable between male and female E3-HL mice, they were greater in male versus female E3-TR mice (*F*(1, 18) = 66.4375, *t* = 8.151, *p* < 0.0001).

There also were effects of model (*F*(1, 33) = 28.6295 *p* < 0.0001) and sex (*F*(1, 33) = 20.3298 *p* < 0.0001) on plasma apoE levels and a sex ×model interaction (*F*(1, 33) = 20.7279 *p* < 0.0001) (Fig. 5B). Plasma apoE levels did not differ between E3-HL and E3-TR male mice but were four-fold higher in E3-HL female than in E3-HL male (*F*(1, 15) = 17.7123, *t* = 4.209, *p* < 0.0008) or E3-TR female mice (*F*(1, 16) = 24.4428, *t* = 4.944, *p* < 0.0001).

While in E3-HL mice there was no sex difference in liver weights (Fig. 5B), males had a larger body weight than females (Suppl. Fig. 1A). As a result, the body weight/liver weight ratio was larger in E3-HL males compared to females (Suppl. Fig. 1B). These data show that larger mice do not necessarily have a larger liver.

### Correlations between plasma apoE levels, behavioral and cognitive measures

**Figure 6:**
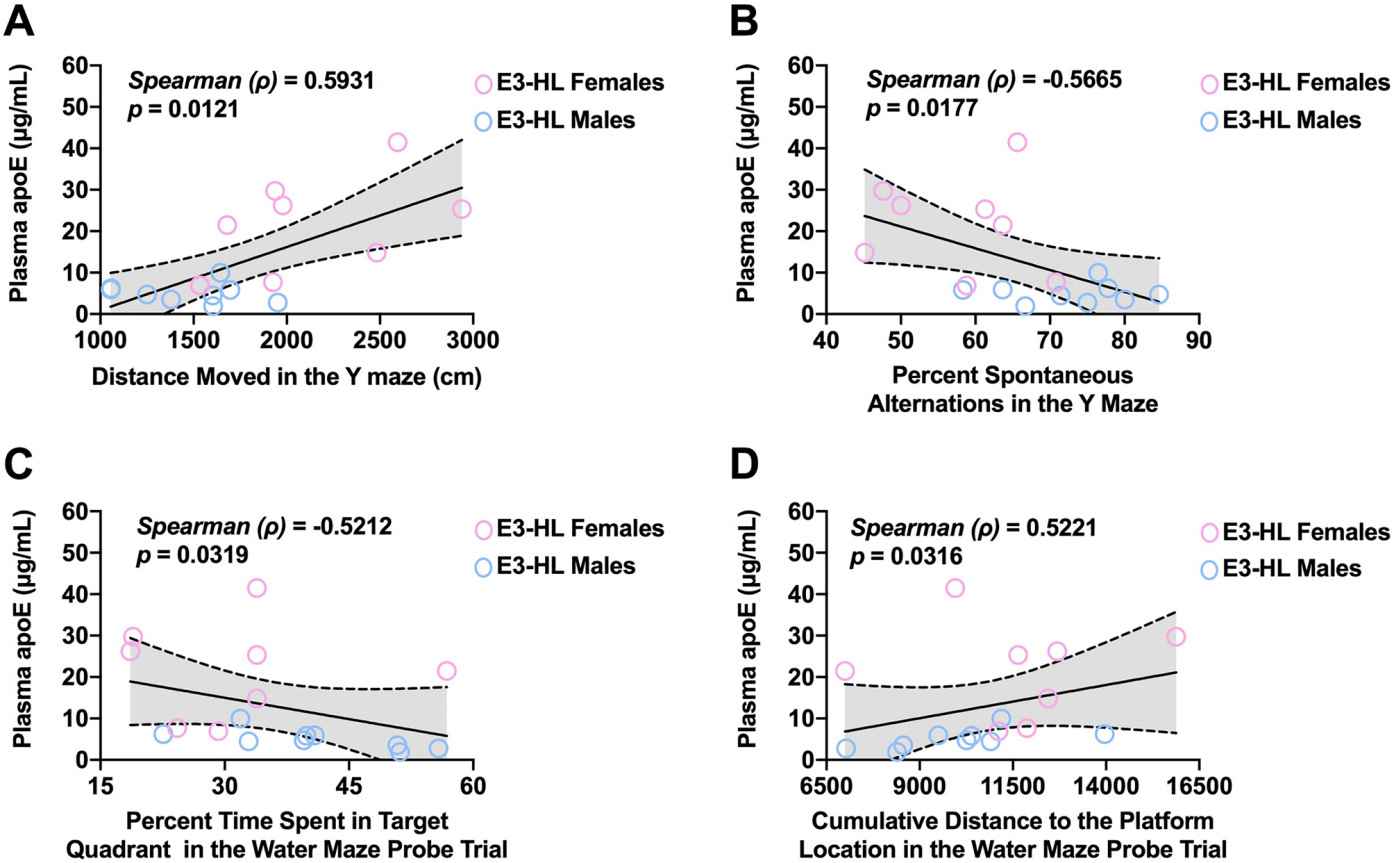
**(A)** In E3-HL mice, plasma apoE levels were positively correlated with activity levels in the Y maze. (**B)** Plasma apoE levels were negatively correlated with spontaneous alternation in the Y maze. (**C)** Plasma apoE levels were negatively correlated with time spent in the target quadrant. (**D)** Plasma apoE levels were positively correlated with cumulative distance to the platform location. In E3-TR mice these correlations were not detected.

In E3-HL mice, plasma apoE levels were positively correlated with activity levels in the Y maze (Fig. 6A). However, in E3-HL mice, plasma apoE levels were negatively correlated with cognitive measures. More specifically, plasma apoE levels were negatively correlated with hippocampus-dependent spontaneous alternation in the Y maze (Fig. 6B), and spatial memory retention in the water maze probe trial; plasma apoE levels were also negatively correlated with time spent in the target quadrant (Fig. 6C) driven by male E3-HL mice (*r* = −0.7084, *p* = 0.0327 (Pearson)), and positively correlated with cumulative distance to the platform location (Fig. 6D). A higher cumulative distance to the platform location reflects worse cognitive performance.

**Figure 7:**
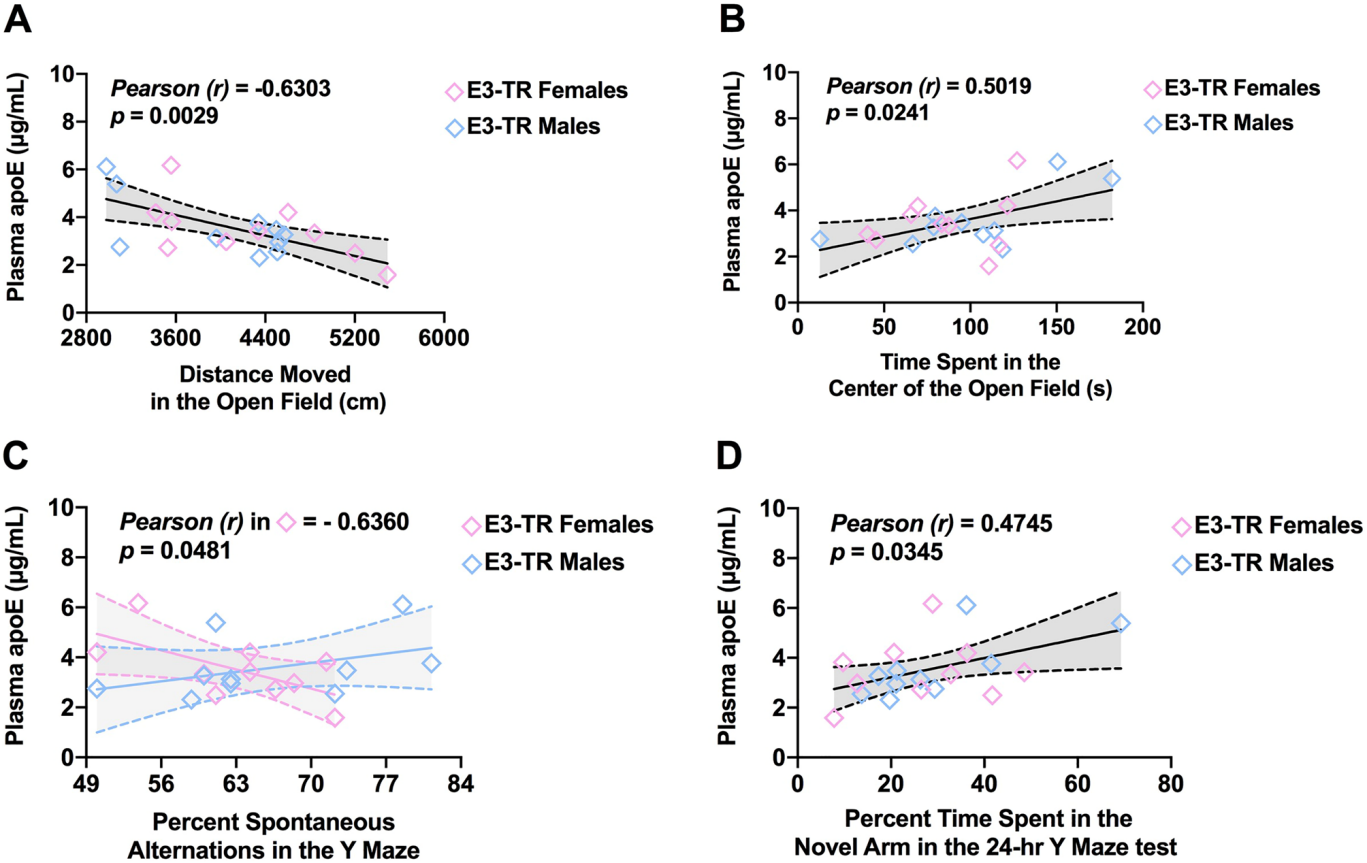
**(A)** In E3-TR mice, plasma apoE levels were negatively correlated with activity levels in the open field. (**B)** In E3-TR mice, plasma apoE levels were positively correlated with time spent in the center of the open field. (**C)** In E3-TR female mice, plasma apoE levels were negatively correlated with spontaneous alternation in the Y maze. (**D)** In E3-TR mice, there was a positive relationship between plasma apoE levels and time spent in novel arms of the spatial Y maze. In E3-HL mice these correlations were absent.

In the E3-TR mice, plasma apoE levels were negatively correlated with activity levels in the open field (Fig. 7A). In contrast, plasma apoE levels were positively correlated with reduced measures of anxiety (i.e., increased time spent in the center) in the open field (Fig. 7B). Similar to the observation in E3-HL mice, plasma apoE levels were negatively correlated with spontaneous alternation in the Y maze in E3-TR female mice (Fig. 7C). However, in contrast to E3-HL mice, this relationship in E3-TR mice was sex-dependent and not seen in male mice (Fig. 7C). Finally, in E3-TR mice there was a positive relationship between plasma apoE levels and time spent in novel arms of the spatial Y maze (Fig. 7D). Thus, with the exception of the Y maze for which there was a negative correlation between plasma apoE levels and spontaneous alternation in E3-HL mice and E3-TR female mice, the observed relationships between plasma apoE levels and behavioral and cognitive measures were model-dependent. Accounting for the sex of the studied mice, correlations remained only in the E3-TR mice (Supplementary Table 1).

**Figure 8:**
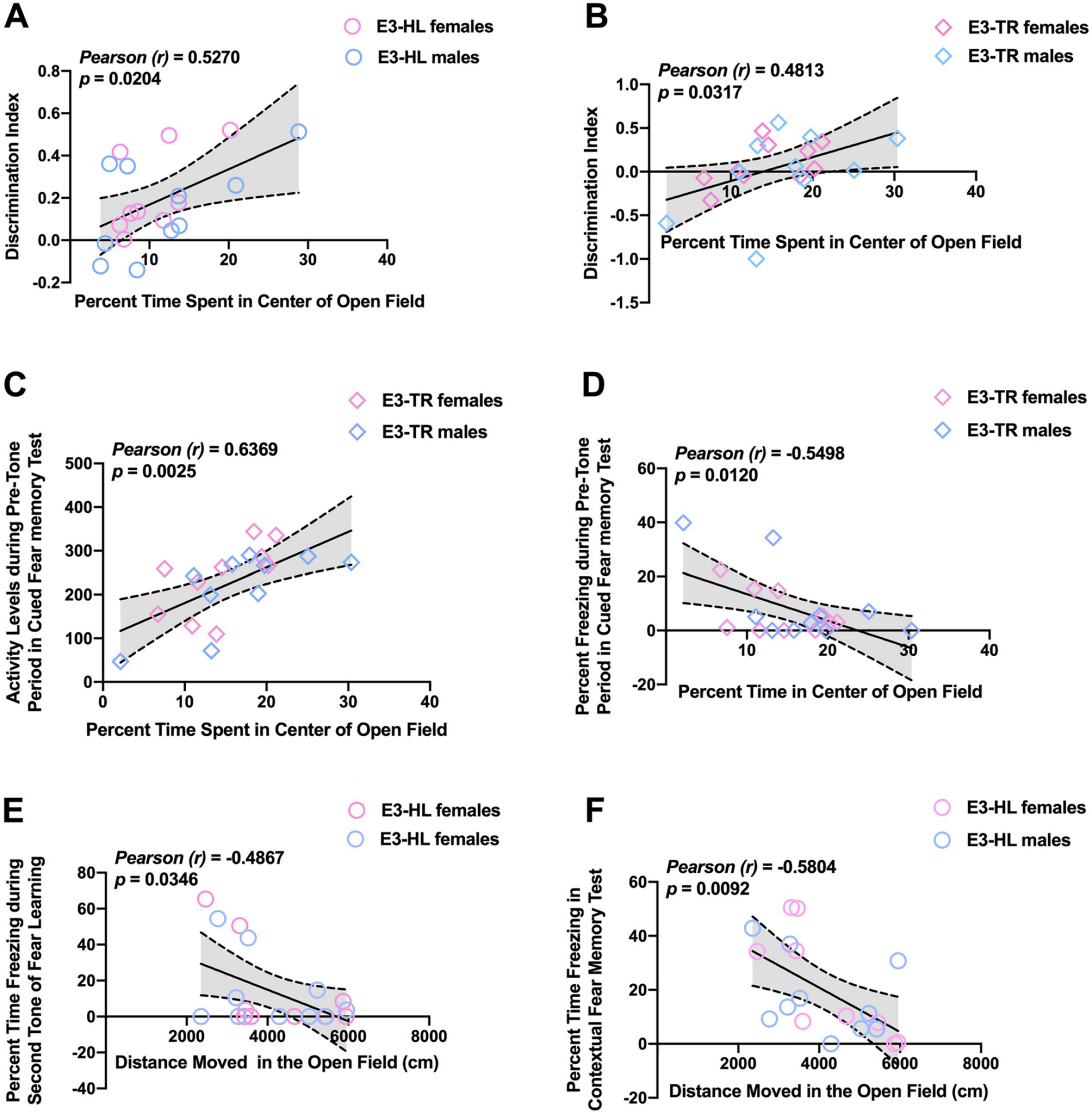
**(A)** In E3-HL mice, percent time spent in the center of the open field positively correlated with the discrimination index in the object recognition test. (**B)** In E3-TR mice, percent time spent in the center of the open field also positively correlated with the discrimination index in the object recognition test. (**C)** In  E3-TR mice, the percent time spent in the center of the open positively correlated with activity levels. (**D)** In E3-TR mice, the percent time spent in the center of the open negatively correlated with percent freezing during the pre-tone period in the cued fear memory test. (**E)** In E3-HL mice, distance moved on the first day of open field testing negatively correlated with the percent time freezing during the second tone during fear learning. (**F)** In E3-HL mice, distance moved on the first day of open field testing negatively correlated with the percent freezing in the contextual fear memory test.

In E3-HL mice, percent time spent in the center of the open field (a measure of reduced anxiety levels) positively correlated with the discrimination index (a measure of object recognition) (Fig. 8A). This relationship was also seen in E3-TR mice (Fig. 8B). Thus, reduced anxiety measures in the open field were correlated with enhanced object recognition. In addition, in E3-TR, but not in E3-HL mice, the percent time spent in the center of the open field positively correlated with activity levels (Fig. 8C) and negatively correlated with percent freezing (Fig. 8D) during the pre-tone period in the cued fear memory test.

In E3-HL mice, distance moved on the first day of open field testing negatively correlated with the percent time freezing during the second tone during fear learning (Fig. 8E) and with the percent freezing in the contextual fear memory test (Fig. 8F). These relationships were not seen in the E3-TR mice. Thus, in E3-HL mice reduced anxiety measures in the open field were correlated with reduced fear learning and contextual fear memory. With the exception of the relationship between measures of anxiety in the open field and object recognition, these relationships were model-dependent.

A principal components factor analysis (PCA) was performed to determine whether plasma apoE levels or liver weight might contribute significantly to performance on behavioral tasks and load on the same factor(s), and to approximate to what extent the distinct measures assess the same underlying abilities in the mice. This analysis revealed six factors with eigenvalues > 1.0 which explained a total of 71.7% of the variance among the measures entered into the model (Table 1). The six factors explained 18.1, 14.5, 14.3, 9.8, 9.2, and 5.8% of the variance, respectively. Liver weight loaded on all six factors and most profoundly on factor 2. Plasma apoE levels loaded on factors 2 through 5 and most profoundly on factors 2 and 5. The directions of the component loading for plasma apoE levels and liver weight were matched for components 2 through 4, but in the opposite direction for component 5. These data indicate that although there is an overlap, liver weight and plasma apoE levels are not necessarily positively related to each other.

**Table 1:** Principal component analysis: component loadings for behavioral measures, plasma apoE, and liver weights.

	1	2	3	4	5	6
Distance moved 1st day of open field	0.851	−0.159			−0.189	
Distance moved 2nd day of open field	0.857	−0.146	0.118	0.214		−0.160
Time in center 1st day of open field	0.159	−0.291		0.840		0.189
Time in center 2nd day of open field	0.192			0.856	0.159	
Distance moved 1st day of object recognition test	0.814	0.364				0.230
Distance moved 2nd day of object	0.739	0.368				0.109
Discrimination index in object recognition test	0.247	0.165		0.252		0.708
Mean latency visible platform training	−0.136	0.781				0.126
Mean latency hidden platform training		0.182	−0.310		0.725	
Mean swim speed visible platform training	0.401	0.399	0.171	0.420	−0.408	−0.272
Crossing platform location in water maze probe trial			0.911			0.110
Percent time in target quadrant in water maze probe trial		−0.102	0.923			
Cumulative distance to the platform in water maze probe trial			−0.890			
Latency to reach the platform location in the water maze probe trial		0.200	−0.677		0.186	0.110
Motion during the baseline period during fear learning	0.576	0.260	−0.121	0.219		0.285
Freezing during the contextual fear memory test	−0.492	−0.659				
Freezing during the cued fear memory test	−0.136	−0.830				
Distance moved in the Y maze	0.343	−0.830				
Percent spontaneous alternation in the Y maze	0.343				−0.735	
Percent time in the novel arm in the 24 h Y maze	0.308		0.147	0.104	0.705	
Percent entries in the novel arm in the 24 h Y maze	−0.303	0.127	0.209	0.617		0.429
Plasma apoE levels		0.607	−0.255	−0.131	−0.452	
Liver weights	0.180	0.604	−0.164	−0.142	0.162	0.192

The verimax rotated matrix was used to interpret the factor loadings. This analysis revealed five factors with eigenvalues > 1.0, which explained a total of 71.7% of the variance among the measures entered into the model. The six factors explained 18.1, 14.5, 14.3, 9.8, 9.2, and 5.8 % of the variance, respectively. For additional details, see main text.

Liver weight, activity levels in the open field without and with objects, swim speeds, time to locate the visible platform in the water maze, distance moved prior to the first tone during fear learning in the fear conditioning test, contextual and cued fear memory, activity levels and spontaneous alternation in the Y maze, and novel arm entries all loaded on factor 1, indicating a common underlying ability being measured by all of these tests. The directions of the component loadings in Factor 1 were such that increasing liver weight increased activity levels in the open field, water maze, fear conditioning test, and Y maze, increased ability to locate the visible platform in the water maze, increased spontaneous alternation in the Y maze and increased the discrimination index in the object recognition test, but decreased contextual and cued fear memory and cognitive performance novel arm measures in the 24 h Y maze. Hence, a larger liver was consistently associated with increased activity levels in different behavioral tests.

Plasma apoE levels and liver weight, activity levels in the open field without and with objects, swim speeds, time to locate the visible and hidden platform and spatial memory retention in the water maze, distance moved prior to the first tone during fear learning in the fear conditioning test, contextual and cued fear memory, and novel arm measures in the 24 h Y maze and liver weights loaded on factor 2. Plasma apoE levels and liver weight loaded most heavily on factor 2. The directions of the component loadings in Factor 2 were such that higher plasma apoE levels and liver weight decreased activity levels in the open field without objects, but increased activity levels in the open field with objects, increased anxiety measures in the open field, increased object recognition, increased swim speeds and ability to locate the visible and hidden platform in the water maze, decreased spatial memory retention in the water maze, decreased contextual and cued fear memory, increased the percent time spent in the novel arm but decreased the percent entries in the novel arm in the Y maze.

Plasma apoE levels and liver weight, spatial memory retention in the water maze, and performance in the 24 h Y maze loaded on factor 3. Spatial memory retention in the water maze most heavily loaded on factor 3. The directions of the component loadings in Factor 3 were such that decreasing plasma apoE levels and liver weight increased ability to locate the hidden platform and spatial memory retention in the water maze, increased spontaneous alternation, and increased cognitive performance in the 24 h Y maze.

Distance moved on the second day of open field testing, measures of anxiety in the open field, object recognition, swim speeds during visible platform training in the water maze, distance moved prior to the first tone during fear learning in the fear conditioning test, spontaneous alternation in the Y maze, percent time spent in the novel arm of the 24 h Y maze, plasma apoE levels and liver weight loaded on factor 4. The directions of the component loadings in Factor 4 were such that decreasing plasma apoE levels and liver weight increased the activity levels in the open field, decreased anxiety levels in the open field, increased swim speeds during visible platform training, increased activity levels prior to the first tone during fear learning in the fear conditioning test, increased spontaneous alternation in the Y maze, and increased time spent in the novel arm in the 24 h Y maze.

Also, plasma apoE levels and liver weights, activity levels on the first day of the open field, anxiety measures on the second day of the open field, swim speeds and ability to locate the hidden platform in the water maze, time to locate the platform location in the probe trial, activity and spontaneous alternation in the Y maze, and percent entries in the novel arm of the 24 h Y maze loaded on factor 5. The directions of the component loadings in Factor 5 were such that decreasing plasma apoE levels but increasing liver weights decreased activity levels and increased anxiety measures in the open field, decreased swim speeds during visible platform training and ability to locate the hidden platform, decreased activity levels and increased spontaneous alternation in the Y maze, and decreased the percent entries in the novel arm in the 24 h Y maze.

Liver weight, activity levels of the second day in the open field, measures of anxiety on the first day in the open field, activity levels in the open field containing object, object recognition, swim speeds and ability to locate the visible platform in the water maze, latency to locate the platform in the water maze probe trial, distance moved prior to the first tone during fear learning in the fear conditioning test, and novel arm measures in the 24 h Y maze loaded on factor 6. The directions of the component loadings in Factor 6 were such that higher liver weights decreased activity levels on the second day in the open field, decreased measures of anxiety on the first day of the open field, increased activity levels in the open field containing object, increased object recognition, decreased swim speeds and ability to locate the visible platform in the water maze, increased measures of spatial memory retention in the water maze, increased distance moved prior to the first tone during fear learning in the fear conditioning test, and increased novel arm entries in the 24 h Y maze.

These outcomes of the PCA support a role for both plasma apoE levels and liver size in behavioral and cognitive measures.

### Plasma apoE distribution in lipoparticle fractions

**Figure 9:**
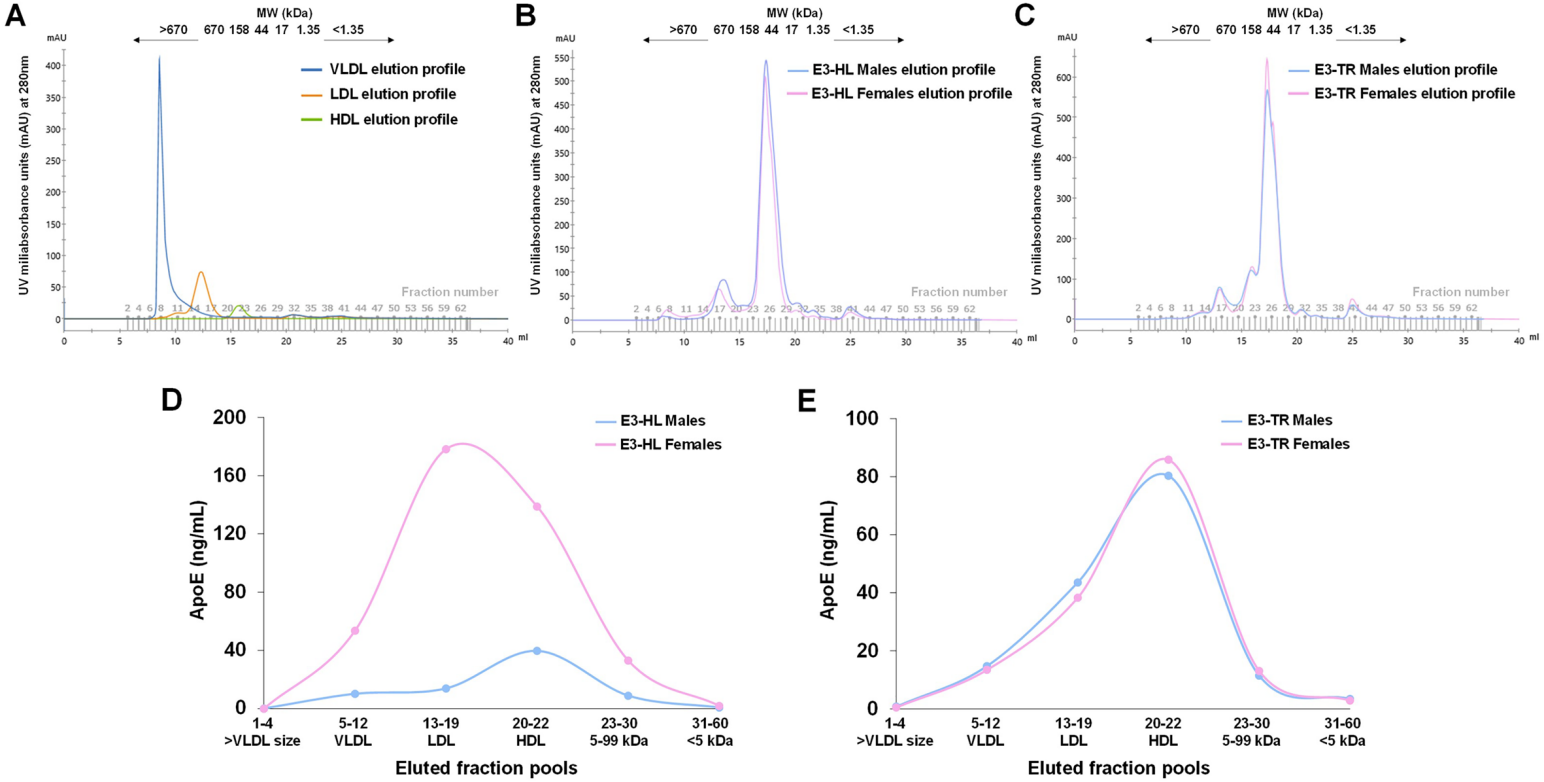
**(A)** Overlap of raw chromatograms of human plasma isolated lipoproteins VLDL, LDL and HDL. (**B,C)** Raw chromatograms of plasma from female and male E3-HL (**B**) and E3-TR mice (**C**). (**D,E)** ApoE elution profile in the six fraction pools obtained after SEC fractionation of plasma from female and male E3-HL (**D**) or E3-TR mice (**E**). Plasma Pool 1: Fractions 1–4 containing molecules larger than VLDL, Pool 2: Fractions 5–12 corresponding to the VLDL eluted fraction, Pool 3: Fractions 13–19 corresponding to the LDL eluted fraction, Pool 4: Fractions 20–22 corresponding to HDL eluted fraction, Pool 5: Fractions 23–30 corresponding to fractions with molecular size molecules ranging between 99 and 5 kDa, Pool 6: Fractions 31–60 corresponding to fractions with lower than 5 kDa proteins.

Due to the identified sex and mouse model-dependent differences in plasma apoE levels, we assessed whether these factors also impacted the distribution of plasma apoE into the lipoparticles VLDL, LDL and HDL. Raw SEC elution profiles including molecular size are presented for commercially acquired isolated plasma VLDL, LDL and HDL (Fig. 9A) and pools of plasma from E3-HL and E3-TR female and male mice (Fig. 9B,C). Following SEC of plasma pools (male versus female E3-HL mice, male versus female E3-TR mice) we observed that the apoE distribution in the six fractionated plasma pools differed between E3-HL female and male mice (Fig. 9D), indicating more apoE in larger lipoparticles in females whereas more apoE appeared associated with smaller lipoparticle size in male mice. No such sex-difference was present in E3-TR mice (Fig. 9E).

Assessment of the percentage of apoE eluting in specific lipoparticle fractions of the total lipoparticle-associated apoE confirmed that apoE in male E3-HL mice was predominantly found in the HDL fraction with 1.63-fold more HDL-associated apoE compared to female mice. Inversely, apoE was mainly associated with LDL in the E3-HL mice with 2.18-fold more LDL-associated apoE than in make mice (Table 2). In the E3-TR mice, the apoE was mainly distributed to the HDL faction with no difference between male and female mice (Table 2).

**Table 2:** Lipoparticle-associated apoE distribution in VLDL, LDL and HDL fractions.

Mouse model	Sex	% apoE in the VLDL fraction	% apoE in the LDL fraction	% apoE in the HDL fraction	apoE ratio in LDL/HDL fractions
E3-HL	Male	16	22	62	0.35
	Female	14	48	38	1.28
E3-TR	Male	11	31	58	0.54
	Female	10	28	62	0.45

*E3-HL* *APOE* ε3/ε3 humanized liver mice, *E3-TR* *APOE* ε3/ε3 targeted replacement mice, *VLDL* very low density lipoprotein, *LDL* low density lipoprotein, *HDL* high density lipoprotein, *apoE* apolipoprotein E.

## Discussion

Varying based on ethnicity, between 50 and 74% of reported control subjects were *APOE*ε3 homozygous (http://www.alzgene.org/meta.asp?geneID=83) in clinical AD-association studies. The *APOE*ε3 variant is considered ‘neutral’ in relation to the risk of developing AD, yet between 26 and 54% of AD patients are homozygous for this genotype. Low plasma apoE levels were previously directly associated with a higher risk of developing AD and all dementias^14^ and we have previously described a significant sex-dependent association specifically between the plasma apoE3 isoform levels and plasma glucose levels^45^. This association in turn was associated with brain glucose metabolism in cognitively healthy *APOE*ε3/ε4-carriers^18^. We have also reported that FRGN mice with humanized livers of the *APOE*ε4/ε4 genotype in comparison to mice with *APOE*ε2/ε3 livers exhibited lower endogenous mouse cortical and to some extent also hippocampal apoE levels^19^. The liver-associated changes in brain apoE concentrations were linked to altered levels of various markers of synaptic integrity, insulin signaling and neuroinflammation. Importantly, a potential association between plasma apoE levels and behavior and memory outcomes in the FRGN humanized liver mouse model was so far unexplored. In the current study we therefore addressed a potential link between peripheral, plasma liver-derived apoE levels and behavior and memory phenotypes in two different mouse models; the FRGN humanized liver mice (E3-HL) and the APOE-TR (E3-TR) mice expressing the human ‘AD-neutral’ *APOE*ε3 variant. Whereas the E3-TR mice express *APOE*ε3 in place of the rodent *Apoe* throughout the body under the control of the mouse *apoe* promotor, the FRGN humanized liver mice only express human *APOE*ε3 in the humanized liver under the control of endogenous human regulatory mechanisms but rodent *Apoe* outside of the liver. The FRGN humanized liver mice were described to exhibit a human-like plasma lipid profile^46^ which makes them a superior model for translational studies into hepatic lipid and drug metabolism^46–48^.

The results of the current study showed model-dependent differences in various behavior (i.e., activity and anxiety levels) and cognitive (*i.e.*, spatial memory retention in the water maze and fear learning and contextual and cued fear memory), measures between male and female mice. However, plasma apoE levels varied between the sexes only in the E3-HL mice in which females had the highest plasma apoE levels. In the water maze probe trial, HL-E3 mice showed reduced spatial memory retention. The HL-E3 female mice crossed the platform location less than female TR-E3 mice and the latency to first reach the platform location was higher in HL-E3 than TR-E3 mice. The fact that this cognitive phenotype was driven by HL-E3 female mice is remarkable considering that they had much higher plasma apoE levels than HL-E3 males and E3-TR mice. Furthermore, in the open field containing objects, E3-HL mice moved more in the presence of objects than E3-TR mice, suggesting a beneficial exploratory drive and response to novelty. However, compared to E3-TR mice, E3-HL mice showed reduced fear learning and contextual and cued fear memory. The fact that both contextual and cued fear memory were lower indicates that cognitive phenotypes are not limited to the hippocampus.

An effect of model was seen for some hippocampus-dependent spatial memory retention in the water maze probe trial and contextual fear conditioning, and there was a trend towards an effect of model in the hippocampus-dependent spatial Y maze. In contrast, no effect of model was seen for hippocampus-dependent spatial habituation learning, object recognition, ability to locate the hidden platform in the water maze, or spontaneous alternation in the Y maze. These results indicate that while these tests involve the hippocampus, they might differ in various aspects causing there to be an effect of model in some but not other cognitive tests. In addition, there was an effect of model for hippocampus-independent cued fear memory. These data indicate that hippocampal involvement is not necessarily needed or sufficient to detect an effect of model.

Reduced anxiety measures in the open field were correlated with enhanced object recognition in E3-HL and E3-TR mice. Conceptually this is not surprising. Neophobia interferes with exploring novel object and reduced anxiety measures would be expected to do the opposite. This results is also consistent with the results of the PCA analysis showing that measures of anxiety in the open field and object recognition both loaded on factor 4.

With the exception of this positive relationship between measures of anxiety in the open field and object recognition that was seen in both mouse models, the relationships between anxiety or activity levels and cognitive measure were model-dependent. In E3-TR, but not in E3-HL, mice the percent time spent in the center of the open positively correlated with activity levels and negatively correlated with percent freezing during the pre-tone period in the cued fear memory test. In contrast, in E3-HL, but not in E3-TR, mice distance moved on the first day of open field testing negatively correlated with the percent time freezing during the second tone during fear learning and with the percent freezing in the contextual fear memory test. These data indicate that the relationships between measures of anxiety and activity levels with cognitive measures are predominantly model-dependent.

In our study, the liver was profoundly larger in E3-HL than E3-TR mice. This finding is consistent with earlier studies involving mice with livers repopulated with human hepatocytes^49^. While the ratio of liver-to-body size is normally tightly controlled, human hepatocytes do not respond to fibroblast growth factor 15 (FGF15) generated in the mouse intestine, resulting in an enlargement of the bile acid pool^49^. Transgenic insertion of the human homolog of FGF15, FGF19, in the human hepatocytes, reduced the liver size to that typically seen in mice^49^. Administration of recombinant FGF19 to humanized liver mice also reduced the liver size^46^. Transgenic expression and administration of recombinant FGF19 also reduced the enlargement of the bile acid pool which in humanized liver mice might mediate brain-related phenotypes. While the brain can generate bile acids, the majority of bile acids in the brain is taken up from the circulation^50^. The gut microbiome is involved in the regulation and synthesis of bile acids and the gut-liver-brain axis might play an important role in mediating the behavioral phenotypes of humanized liver mice.

The relationships revealed between plasma apoE levels and behavioral and cognitive measures in E3-HL and E3-TR mice, support a role of the liver in the phenotypes seen. Remarkably, in E3-HL mice plasma apoE levels were positively related to activity levels but negatively related to cognitive measures. In contrast to what was observed in E3-HL mice, plasma apoE levels were negatively related to activity levels in E3-TR mice. As in both models we found a negative relationship with cognitive performance in the Y maze (spontaneous alteration), these data suggest that increased activity levels in a novel environment are not necessarily related to improved cognitive performance. In the E3-TR mice, we documented a positive relationship between plasma apoE levels and reduced anxiety measures in the open field. This result is very much in line with the anxiety phenotype seen in apoE-deficient mice^7^. However, also apoE isoforms might be of importance as E2-TR and E4-TR compared to E3-TR mice show higher anxiety levels while plasma apoE levels are typically higher in E2 than E3 and E4 mice^51,52^.

With regard to cognitive measures, the pattern seen in E3-TR mice was more complicated than in E3-HL mice. While a negative relationship was seen between plasma apoE levels in TR-E3 female mice, this was not seen in TR-E3 male mice. In addition, in E3-TR mice plasma apoE levels were positively correlated to cognitive performance in the 24-h spatial Y maze. Although performance in both Y mazes is hippocampus-dependent, the 24-h spatial Y maze involves long-term spatial memory retention. With the exception of spontaneous alternation in the Y maze, for which a negative relationship was seen between plasma apoE levels and spontaneous alternation in E3-TR females and E3-HL females and males, these relationships were model-dependent. The negative relationship between plasma apoE levels and cognitive measures suggests that the notion that reduced plasma apoE levels in E4 carriers as compared to non-E4 carriers might be related to reduced cognitive performance^53^ and reduced hippocampal volumes^54,^ is too simplistic. The pattern previously seen is consistent with a dominant negative effect of E4, as compared to E3, for specific cognitive phenotypes, when both are purposely expressed at equal levels in brain on a murine apoE deficient background and compared to mice lacking murine apoE in and outside the brain^6^. Also, as indicated by results from a previous clinical study showing that higher levels of HDL-associated apoE were associated with better cognition^55^ the distribution of plasma apoE into specific lipoparticle fractions may be of overall importance to cognitive performance and behavior. In our study, the E3-HL mice exhibited a sex-dependent difference in the distribution of apoE into HDLs and LDLs with male mice exhibiting more HDL-associated apoE than the female mice.

This first proof of concept study has the following limitation; Due to the nature of generating and maintaining humanized liver mice, the diet and treatments in the drinking water that the E3-HL mice received but E3-TR mice did not, we cannot exclude that those differences might have contributed to some of the behavioral and cognitive phenotypes seen in this study. As for various behavioral and cognitive measures there were model × sex interactions, those environmental differences do not seem sufficient to induce behavioral and cognitive phenotypes. In addition, the results of the PCA show that plasma apoE levels and liver size load on the same factors as distinct behavioral and cognitive measures, supporting that plasma apoE levels and liver size contribute to the behavioral and cognitive measures.

While all groups showed comparable object recognition, the mice performed relatively poorly on this test. This might be because of stress due to testing outside of the home cage. In this context, the development of an object recognition test in the home cage and soiling novel objects with bedding from the home cage^56^ is interesting. Although using olfactory cues from the home cage and testing in the home cage might diminish the novelty aspect, this testing scenario likely enhances exploration of the novel objects.

## Conclusion

The results of this study suggest model- and sex-specific associations between plasma apoE, the distribution of apoE into HDL/LDL lipoparticles and behavior and memory phenotypes in FRGN humanized livers and *APOE*-targeted replacement mice expressing the ‘AD-neutral’ *APOE*ε3 variant. These data confirm that there are mouse-model dependent associations between plasma apoE levels and behavioral and cognitive performance and that a humanized liver and related plasma apoE levels are sufficient to induce mouse behavioral and cognitive phenotypes. The PCA results show that plasma apoE levels and liver size load on the same factors as distinct behavioral and cognitive measures, supporting that plasma apoE levels and liver size contribute to the behavioral and cognitive measures. Future efforts are warranted to compare behavioral and cognitive phenotypes in humanized liver mice involving donors with distinct *APOE* genotypes to determine whether the phenotypes seen in the current study are apoE isoform-dependent.

## Supplementary Information

Supplementary Information.

## Data Availability

Upon request following contacting Drs. Raber or Nielsen, the raw data supporting the conclusions of this article will be made available by the authors, without undue reservation.

## Abbreviations

AD: Alzheimer’s disease
Aβ: Amyloid β
ApoE: Apolipoprotein E
BBB: Blood–brain-barrier
CS: Conditioned stimulus
FGF15: Fibroblast growth factor 15
FRGN: *Fah*−/−, *Rag2*−/−,* Il2rg*−/− mice on the non-obese diabetic (NOD) background
HL: Human liver
HRP: Horse radish peroxidase
NFDMP: Nonfat dried milk powder
NOD: Non-obese diabetic
SMX/TMP: Sulfamethoxazole/trimethoprim
TR: Targeted replacement
