# Supplementary material for: Behavioral and cognitive performance of humanized APOEε3/ε3 liver mice in relation to plasma apolipoprotein E levels
Source: Sci Rep. 2023 Jan 31;13:1728. doi: 10.1038/s41598-023-28165-3 (PMC9889814; doi:10.1038/s41598-023-28165-3)
Supplement: Supplementary file 1 — Supplementary Information. [file 41598_2023_28165_MOESM1_ESM.docx]

**Supplementary Figures, Figure Legends, and Table.**

**Supplementary Figure 1.** Body weight and body-to-liver ratio of female and male E3-HL mice. **A.** Male E3-HL (*n* = 10, 28.8 ± 1.12) mice weighed 3.2g more than female mice (*n* = 9, 25.6 ± 0.71). Statistical significance was assessed using student’s *t*-test *(t* = 2.303, df = 17, **p* = 0.0342). **B.** Male E3-HL (*n* = 10, 10.6 ± 0.32) mice had a 1.1-fold higher body-to-liver weight ratio compared to female mice (*n* = 9, 9.48 ± 0.15). Group comparison was performed using student’s *t*-test *(t* = 3.097, df = 17, ***p* =0.0066). Data is shown as mean ± SEM.


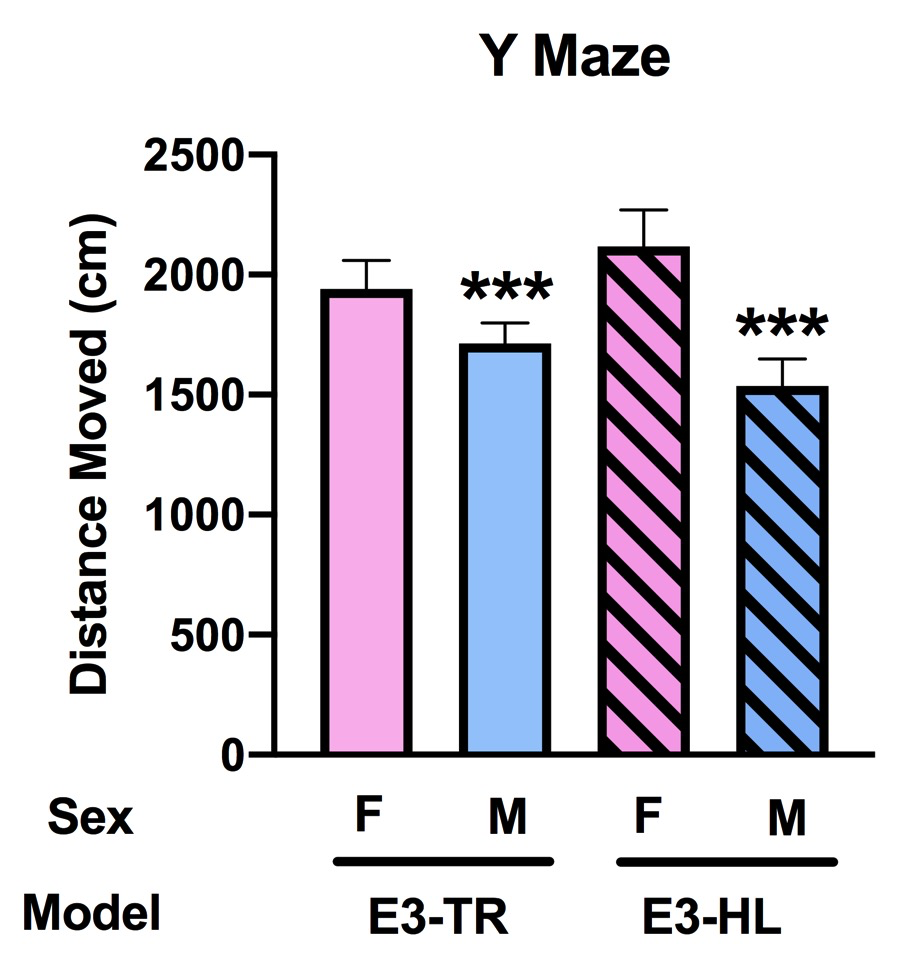


**Supplementary Figure 2.** Distance moved in the Y maze. There was an effect of sex, with higher activity levels in females than males. ****p* = 0.0041. Data is shown as mean ± SEM.

**Supplementary Table 1:** Partial correlations between plasma apoE3 levels with the significant cognitive and behavioural measurements after accounting for the sex of the studied mice

| Mouse model | Behavioural or cognitive measurements | Partial correlation | *p*-value |
| --- | --- | --- | --- |
| E3-HL  (*n* = 17) | Distance Moved in the Y Maze (cm) | *r*(14) = 0.141 | 0.601 |
|  | Percent Spontaneous Alterations in the Y Maze | *r*(14) = – 0.058 | 0.830 |
|  | Percent Time Spent in Target Quadrant in the Water Maze Probe Trial | *r*(14) = – 0.388 | 0.137 |
|  | Cumulative Distance to the Platform Location in the Water Maze Probe Trial | *r*(14) = 0.390 | 0.135 |
| E3-TR  (*n* = 20) | Distance Moved in the Open Field (cm) | *r*(17) = – 0.636 | 0.003 |
|  | Time Spent in the Center of the Open Field (s) | *r*(17) = 0.504 | 0.028 |
|  | Percent Time Spent in the Novel Arm in the  24-hr Y Maze test | *r*(17) = 0.474 | 0.040 |

Partial correlations are shown as (*r*(degrees of freedom))
